# New distribution records, first host plant record and DNA barcoding of the Neotropical plume moth *Oidaematophoruspseudotrachyphloeus* Gielis (Lepidoptera, Pterophoridae)

**DOI:** 10.3897/BDJ.9.e77167

**Published:** 2021-11-24

**Authors:** Héctor A. Vargas

**Affiliations:** 1 Universidad Tarapacá, Facultad de Ciencias Agronómicas, Departamento de Recursos Ambientales, Arica, Chile Universidad Tarapacá, Facultad de Ciencias Agronómicas, Departamento de Recursos Ambientales Arica Chile

**Keywords:** Asteraceae, Atacama Desert, Chile, folivorous larvae, mitochondrial DNA

## Abstract

**Background:**

*Oidaematophoruspseudotrachyphloeus* Gielis, 2011 (Lepidoptera, Pterophoridae) is a little-known Neotropical plume moth previously recorded in Ecuador, Peru and Argentina. Its host plants and DNA barcodes are unknown.

**New information:**

*Oidaematophoruspseudotrachyphloeus* is recorded for the first time from Chile, based on six specimens from the Azapa Valley (Arica Province) and two from Guayacán (Coquimbo Province). Those from the Azapa Valley were reared from folivorous larvae collected on *Ambrosiacumanensis* Kunth (Asteraceae), representing the first host plant record for this plume moth. The first DNA barcode sequences of *O.pseudotrachyphloeus* are provided and used to explore relationships with congenerics.

## Introduction

*Oidaematophorus* Wallengren, 1862 (Lepidoptera, Pterophoridae, Pterophorinae, Oidaematophorini) is a widespread genus of plume moths with 24 species described (Gielis 2003, Matthews 2005, Gielis 2011a, Gielis 2011b, Hernandez et al. 2014). Species of this genus are recognised by forewing venation with R1 absent, R2, R3, R4 and R5 separate, Cu1 from the posterior angle of the discal cell and Cu2 from the discal cell, mid-leg with scale bristles at base of spur pairs and female genitalia with bell- or widened funnel-shaped antrum (Gielis 2011a). Host plants are mainly Asteraceae, but feeding on representatives of Onagraceae and Plantaginaceae has been also documented (Matthews and Lott 2005).

Six species of *Oidaematophorus* were listed by Gielis (2011a) from the Neotropical Region. Two species were described subsequently, *O.espeletiae* Hernández, Fuentes, Fajardo & Matthews, 2014 from Colombia (Hernandez et al. 2014) and *O.androsensis* Matthews, 2019 from the Bahamas (Matthews et al. 2019), while two others, *O.papallacta* (Gielis, 2011) and *O.praenigratus* (Meyrick, 1921), were transferred from *Hellinsia* Tutt, 1905 by Gielis (2014) and Ustjuzhanin et al. (2021), respectively.

Hitherto, *O.pseudotrachyphloeus* Gielis, 2011 is reported in Ecuador, Peru and Argentina (Gielis 2011a, Ustjuzhanin et al. 2021). The first records from Chile are reported here. This discovery adds two new localities, provides the first host plant record and allows the sequencing of the first DNA barcodes of this little-known Neotropical plume moth.

## Materials and methods

Plume moths were reared from larvae collected on *Ambrosiacumanensis* Kunth (Asteraceae) in the Azapa Valley, Arica Province, northern Chile. Eight larvae were collected in July 2017 and placed in plastic vials with leaves of *A.cumanensis* and a paper towel at the bottom. Additional leaves were provided until the larvae completed feeding and pupated, about ten days after collection. Before pupation, two larvae were placed in 95% ethanol at -20°C until DNA extraction. Adults emerged about twelve days after pupation in August 2017. A search for additional conspecifics from Chile was performed in the Colección Entomológica de la Universidad de Tarapacá (IDEA), Arica, Chile. The abdomen of each specimen was removed and placed in 10% potassium hydroxide (KOH) for a few minutes for genitalia dissection, stained with Chlorazol black and Eosin and mounted on slides with Euparal. Images of the genitalia were captured with a Sony CyberShot DSC-HX200V digital camera attached to a Leica M125 stereomicroscope. The identification of the specimens was based on comparisons with the original description of *O.pseudotrachyphloeus* provided by Gielis (2011a) and photographs of the genitalia slide of the male holotype deposited in the Museo de Historia Natural, Universidad Nacional Mayor San Marcos, Lima, Peru (MUSM). The distribution map was generated using SimpleMappr (Shorthouse 2010).

Genomic DNA was extracted from two larvae following the procedures described in Huanca-Mamani et al. (2015). DNA purification, PCR amplification and sequencing of the barcode fragment (Hebert et al. 2003), with the primers LCO-1490 and HCO-2198 (Folmer et al. 1994), were performed in Macrogen Inc. (Seoul, South Korea), following the PCR programme described in Escobar-Suárez et al. (2017). Additional sequences (Table 1) with species identification and 658 base pair length were downloaded from BOLD (Ratnasingham and Hebert 2007) for analysis, including congenerics and representatives of other genera of the tribe Oidaematophorini: *Adaina* Tutt, 1905, *Emmelina* Tutt, 1905 and *Hellinsia* Tutt, 1905. These three genera were used as the outgroup because they are closely related to *Oidaematophorus* (Gielis 1993). Unfortunately, sequences of *Oidaematophorus* currently available in public databases are restricted to Nearctic and Palearctic representatives. The sequences were aligned by the ClustalW method and sequence divergence was estimated by the Kimura 2-Parameter (K2P) method in MEGA X (Kumar et al. 2018). Substitution saturation was estimated with the Xia test (Xia et al. 2003) in DAMBE 7 (Xia 2018) to assess the presence of phylogenetic information. A phylogenetic tree was inferred through a Maximum Likelihood (ML) analysis using IQ-TREE 1.6.12 (Nguyen et al. 2015) in the web interface W-IQ-TREE (Trifinopoulos et al. 2016). Data were partitioned to codon position and TNe+I, F81+F and HKY+F+G4 were selected as the best-fit models for 1st, 2nd and 3rd partitions, respectively, in ModelFinder (Kalyaanamoorthy et al. 2017). Branch support was calculated with 1,000 replications of ultrafast bootstrap (UFBoot, Hoang et al. 2017). The unrooted tree was visualised in FigTree (Rambaut 2014) to root on the representative of *Hellinsia*, following Gielis (1993).

## Taxon treatments

### Oidaematophorus pseudotrachyphloeus

Gielis, 2011

6305A949-1E7D-55D4-B6C2-071DC1A1377C

#### Materials

- **Type status:** Other material. **Occurrence:** sex: female; lifeStage: adult; otherCatalogNumbers: genitalia slide HAV1087; **Taxon:** scientificName: *Oidaematophoruspseudotrachyphloeus*; order: Lepidoptera; family: Pterophoridae; taxonRank: species; scientificNameAuthorship: Gielis, 2011; **Location:** continent: South America; country: Chile; stateProvince: Arica; locality: Azapa Valley km 12; verbatimCoordinates: 18°31’16’’S 70°10’42’’W; **Identification:** identifiedBy: H.A. Vargas; dateIdentified: October 2021; **Event:** samplingProtocol: One female adult emerged August 2017, reared from larva collected on *Ambrosiacumanensis* July 2017; year: 2017; month: August; **Record Level:** type: PhysicalObject; institutionCode: IDEA; basisOfRecord: PreservedSpecimen
- **Type status:** Other material. **Occurrence:** sex: female; lifeStage: adult; otherCatalogNumbers: genitalia slide HAV1090; **Taxon:** scientificName: *Oidaematophoruspseudotrachyphloeus*; order: Lepidoptera; family: Pterophoridae; taxonRank: species; scientificNameAuthorship: Gielis, 2011; **Location:** continent: South America; country: Chile; stateProvince: Arica; locality: Azapa Valley km 12; verbatimCoordinates: 18°31’16’’S 70°10’42’’W; **Identification:** identifiedBy: H.A. Vargas; dateIdentified: October 2021; **Event:** samplingProtocol: One female adult emerged August 2017, reared from larva collected on *Ambrosiacumanensis* July 2017; year: 2017; month: August; **Record Level:** type: PhysicalObject; institutionCode: IDEA; basisOfRecord: PreservedSpecimen
- **Type status:** Other material. **Occurrence:** sex: female; lifeStage: adult; otherCatalogNumbers: genitalia slide HAV1479; **Taxon:** scientificName: *Oidaematophoruspseudotrachyphloeus*; order: Lepidoptera; family: Pterophoridae; taxonRank: species; scientificNameAuthorship: Gielis, 2011; **Location:** continent: South America; country: Chile; stateProvince: Arica; locality: Azapa Valley km 12; verbatimCoordinates: 18°31’16’’S 70°10’42’’W; **Identification:** identifiedBy: H.A. Vargas; dateIdentified: October 2021; **Event:** samplingProtocol: One female adult emerged August 2017, reared from larva collected on *Ambrosiacumanensis* July 2017; year: 2017; month: August; **Record Level:** type: PhysicalObject; institutionCode: IDEA; basisOfRecord: PreservedSpecimen
- **Type status:** Other material. **Occurrence:** sex: male; lifeStage: adult; otherCatalogNumbers: genitalia slide HAV1086; **Taxon:** scientificName: *Oidaematophoruspseudotrachyphloeus*; order: Lepidoptera; family: Pterophoridae; taxonRank: species; scientificNameAuthorship: Gielis, 2011; **Location:** continent: South America; country: Chile; stateProvince: Arica; locality: Azapa Valley km 12; verbatimCoordinates: 18°31’16’’S 70°10’42’’W; **Identification:** identifiedBy: H.A. Vargas; dateIdentified: October 2021; **Event:** samplingProtocol: One male adult emerged August 2017, reared from larva collected on *Ambrosiacumanensis* July 2017; year: 2017; month: August; **Record Level:** type: PhysicalObject; institutionCode: IDEA; basisOfRecord: PreservedSpecimen
- **Type status:** Other material. **Occurrence:** sex: male; lifeStage: adult; otherCatalogNumbers: genitalia slide HAV1089; **Taxon:** scientificName: *Oidaematophoruspseudotrachyphloeus*; order: Lepidoptera; family: Pterophoridae; taxonRank: species; scientificNameAuthorship: Gielis, 2011; **Location:** continent: South America; country: Chile; stateProvince: Arica; locality: Azapa Valley km 12; verbatimCoordinates: 18°31’16’’S 70°10’42’’W; **Identification:** identifiedBy: H.A. Vargas; dateIdentified: October 2021; **Event:** samplingProtocol: One male adult emerged August 2017, reared from larva collected on *Ambrosiacumanensis* July 2017; year: 2017; month: August; **Record Level:** type: PhysicalObject; institutionCode: IDEA; basisOfRecord: PreservedSpecimen
- **Type status:** Other material. **Occurrence:** sex: male; lifeStage: adult; otherCatalogNumbers: genitalia slide HAV1108; **Taxon:** scientificName: *Oidaematophoruspseudotrachyphloeus*; order: Lepidoptera; family: Pterophoridae; taxonRank: species; scientificNameAuthorship: Gielis, 2011; **Location:** continent: South America; country: Chile; stateProvince: Arica; locality: Azapa Valley km 12; verbatimCoordinates: 18°31’16’’S 70°10’42’’W; **Identification:** identifiedBy: H.A. Vargas; dateIdentified: October 2021; **Event:** samplingProtocol: One male adult emerged August 2017, reared from larva collected on *Ambrosiacumanensis* July 2017; year: 2017; month: August; **Record Level:** type: PhysicalObject; institutionCode: IDEA; basisOfRecord: PreservedSpecimen
- **Type status:** Other material. **Occurrence:** sex: female; lifeStage: adult; otherCatalogNumbers: genitalia slide HAV1481; **Taxon:** scientificName: *Oidaematophoruspseudotrachyphloeus*; order: Lepidoptera; family: Pterophoridae; taxonRank: species; scientificNameAuthorship: Gielis, 2011; **Location:** continent: South America; country: Chile; stateProvince: Coquimbo; locality: Guayacán; verbatimCoordinates: 29°57’50’’S 71°20’51’’W; **Identification:** identifiedBy: H.A. Vargas; dateIdentified: October 2021; **Event:** samplingProtocol: One female adult collected with malaise trap; year: 1976; month: September; **Record Level:** type: PhysicalObject; institutionCode: IDEA; basisOfRecord: PreservedSpecimen
- **Type status:** Other material. **Occurrence:** sex: male; lifeStage: adult; otherCatalogNumbers: genitalia slide HAV1480; **Taxon:** scientificName: *Oidaematophoruspseudotrachyphloeus*; order: Lepidoptera; family: Pterophoridae; taxonRank: species; scientificNameAuthorship: Gielis, 2011; **Location:** continent: South America; country: Chile; stateProvince: Coquimbo; locality: Guayacán; verbatimCoordinates: 29°57’50’’S 71°20’51’’W; **Identification:** identifiedBy: H.A. Vargas; dateIdentified: October 2021; **Event:** samplingProtocol: One male adult collected with malaise trap; year: 1976; month: September; **Record Level:** type: PhysicalObject; institutionCode: IDEA; basisOfRecord: PreservedSpecimen

#### Taxonomic identification

**Figure 1.**
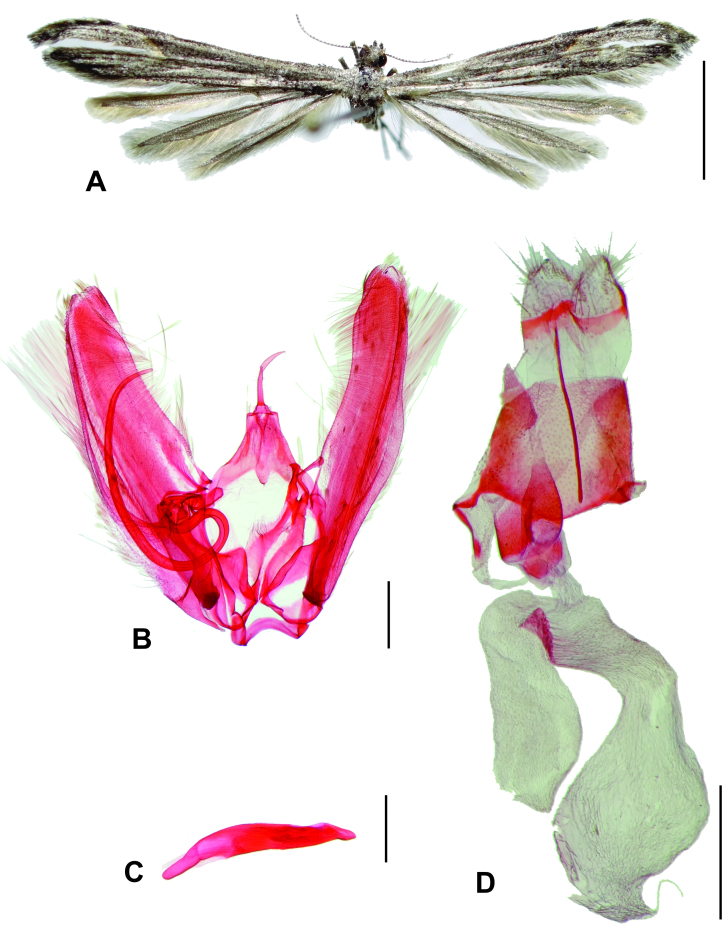
*Oidaematophoruspseudotrachyphloeus* Gielis, 2011 from Chile. **A** Female adult in dorsal view; **B** Male genitalia in ventral view, phallus removed; **C** Phallus in dorsal view; **D** Female genitalia in ventral view. Scale bars 5, 0.5, 0.5, 0.5 mm, respectively.

Six adults (three females, three males) of *O.pseudotrachyphloeus* (Fig. 1) were obtained from the larvae collected on *A.cumanensis* in the Azapa Valley. Two additional conspecifics (one female, one male) from Guayacán (Coquimbo Province) were found in the IDEA collection.

#### DNA barcoding

**Figure 2.**
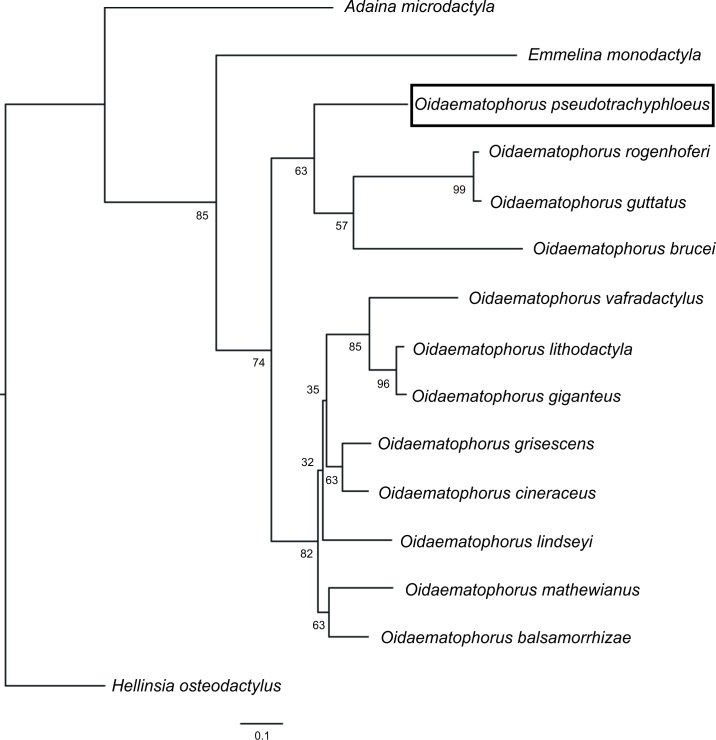
Maximum Likelihood tree of DNA barcodes of *Oidaematophoruspseudotrachyphloeus* Gielis, 2011 and congenerics. Numbers indicate UFBoot support (%) of branches.

Two identical sequences of *O.pseudotrachyphloeus* were obtained (GenBank accessions OK510535, OK510536), which represent the first DNA barcodes for this species. The lowest divergence (9.6% K2P) was with *O.balsamorrhizae* McDunnough, 1939 and *O.cineraceus* (Fish, 1881). *Oidaematophorus* was recovered as a monophyletic group in the ML analysis, but with low UFBoot support (Fig. 2). The relationships of *O.pseudotrachyphloeus* with congenerics were not well resolved.

## Discussion

**Figure 3.**
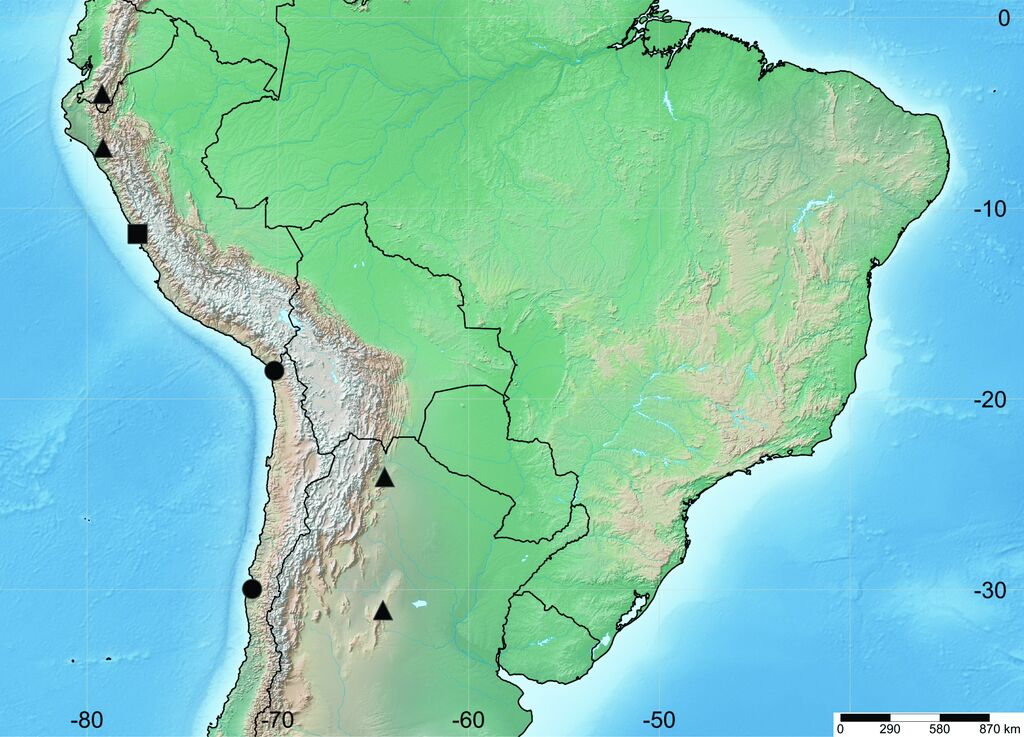
Geographic distribution of *Oidaematophoruspseudotrachyphloeus* Gielis, 2011 in South America. Square (type locality) and triangles indicate previous records, circles indicate new records from Chile.

Based on previous records of *O.pseudotrachyphloeus* (Gielis 2011a, Ustjuzhanin et al. 2021), the northern limit of its geographic distribution is found in Loja (4° 00’ 30’’ S; 79° 12’ 42’’ W; 2030 m a.s.l.), in the Andes of southern Ecuador and the southern limit is east of the Andes in Córdoba (31° 04’ 42’’ S; 64° 30’ 11’’ W; 1000 m a.s.l.) , central Argentina, while the type locality, Reserva Nacional Lomas de Lachay (11° 21’ 00’’ S; 77° 21’ 00’’ W; 290 m a.s.l.), Lima, central Peru, represents the southernmost occurrence west of the Andes. The specimens reported here are the first records of *O.pseudotrachyphloeus* in Chile. Remarkably, the record from the Azapa Valley (18° 31’ 16’’ S; 70° 10’ 42’’ W; 260 m a.s.l.) reveals that the species is able to breed in the extremely arid Atacama Desert and the record from Guayacán (29° 57’ 50’’ S; 71° 20’ 51’’ W; 30 m a.s.l.) expands the range of this plume moth by more than 2000 km to the south along the western margin of South America, exceeding the southern limit of the Atacama Desert (Fig. 3).

*Ambrosiacumanensis*, erroneously cited as *A.peruviana* Willd. in the botanical literature (Luebert and Garcia 2020), is the first host plant recorded for *O.pseudotrachyphloeus*. This discovery agrees with the prevalent association of *Oidaematophorus* with plants of the family Asteraceae (Matthews and Lott 2005). *Ambrosiacumanensis* is a widespread Neotropical species introduced in Chile, whose only records in this country are restricted to the Azapa Valley (Arica Province) (Moreira-Muñoz et al. 2016). As *A.cumanensis* is absent in Guayacán (Coquimbo Province), the presence of *O.pseudotrachyphloeus* in this locality suggests that this plume moth would use a different host plant there. The host plant range deserves further attention, as a more detailed knowledge of it could be helpful to understand the geographic distribution of *O.pseudotrachyphloeus*.

Although the ML analysis here presented is based on a single mitochondrial marker, it is promising that sequences of *Oidaematophorus* were clustered as a monophyletic group in agreement with the most recent morphological definition of the genus (Gielis 2011a). However, the UFBoot support (74%) of this monophylum was low (Minh et al. 2013). Furthermore, as *O.pseudotrachyphloeus* was the only exclusively Neotropical species included in the alignment, it is not surprising that its relationships were not resolved. A more detailed taxon sampling with emphasis on Neotropical representatives of the genus and the use of additional molecular markers would be needed to understand better the evolutionary relationships of *O.pseudotrachyphloeus*.

Eight species of *Oidaematophorus* were previously recorded from Chile (Gielis 1991), all of which were later transferred to *Hellinsia*, based on morphological evidence (Gielis 2011a): *H.betsiae* Gielis, 1991, *H.cinerarius* (Philippi, 1864), *H.coquimboicus* Gielis, 1991, *H.grandaevus* (Meyrick, 1931), *H.hololeucos* (Zeller, 1874), *H.mallecoicus* Gielis, 1991, *H.mauleicus* Gielis, 1991 and *H.siskaellus* Gielis, 1991. The discovery of *O.pseudotrachyphloeus* confirms the presence of *Oidaematophorus* in Chile and highlights the importance of surveys for plume moths in the arid environments of the country as suggested by other recent discoveries (Vargas 2020, Vargas et al. 2020, Ustjuzhanin et al. 2021).

## Supplementary Material

XML Treatment for Oidaematophorus pseudotrachyphloeus

## Figures and Tables

**Table 1.** DNA barcode sequences used in the molecular analysis.

Species	BOLD accession	GenBank accession	Country
*Oidaematophorusbalsamorrhizae* McDunnough, 1939	LNAUS2232-13		USA
*Oidaematophorusbrucei* (Fernald, 1898)	BBLWU081-09	HM428463	USA
*Oidaematophoruscineraceus* (Fish, 1881)	LNAUS2275-13		USA
*Oidaematophorusgiganteus* (Mann, 1855)	PHLAD170-11	KX042801	France
*Oidaematophorusgrisescens* (Walsingham, 1880)	LNAUS2312-13		USA
*Oidaematophorusguttatus* (Walsingham, 1880)	LNAUS2276-13		USA
*Oidaematophoruslindseyi* McDunnough, 1923	LPMNB536-09	KM550521	Canada
*Oidaematophoruslithodactyla* (Treitschke, 1833)	LEATE533-13		Italy
*Oidaematophorusmathewianus* (Zeller, 1874)	JSJUL1684-11	KT126373	Canada
*Oidaematophoruspseudotrachyphloeus* Gielis, 2011		OK510535	Chile
*Oidaematophorusrogenhoferi* (Mann, 1871)	LEALT007-16	MG522712	Russia
*Oidaematophorusvafradactylus* Svensson, 1966	LEFIL230-10	KT782517	Estonia
*Adainamicrodactyla* (Hübner, [1813])	ABOLA573-14		Austria
*Emmelinamonodactyla* Linneaus, 1758	FBLMT634-09	GU706791	Germany
*Hellinsiaosteodactylus* (Zeller, 1841)	ABOLA920-15		Austria
